# Low Light Facilitates Cyclic Electron Flows around PSI to Assist PSII against High Temperature Stress

**DOI:** 10.3390/plants11243537

**Published:** 2022-12-15

**Authors:** Yongjiang Sun, Qi Wang, Huijie Xiao, Jin Cheng

**Affiliations:** 1 Key Laboratory of Forest Silviculture and Conservation of the Ministry of Education, College of Forestry, Beijing Forestry University, Beijing 100083, China; 2 School of Soil and Water Conservation, Beijing Forestry University, Beijing 100083, China; 3 Beijing Key Laboratory of Ornamental Plants Germplasm Innovation and Molecular Breeding, College of Biological Sciences and Biotechnology, Beijing Forestry University, Beijing 100083, China

**Keywords:** heat stress, low light, cyclic electron flows around PSI, grapevine

## Abstract

Photosystem II (PSII) of grapevine leaves is easily damaged under heat stress, but no such injury is observed when the leaves are heated in low light. To elucidate the mechanisms, we compared the photosynthetic characteristics of grapevine seedlings under heat treatments (42 °C) for 4 h in the dark or low light (200 μmol m^−2^ s^−1^). At 42 °C in the dark, the PSII maximum quantum yield (*Fv*/*Fm*) decreased significantly with the increase in time but did not change much in low light. The JIP (chlorophyll a fluorescence rise kinetics) test results showed that low light significantly alleviated the damage to the oxygen evolving complexes (OECs; the K-step was less visible) by heat stress. Further, in the presence of de novo D1 protein synthesis inhibitor chloramphenicol, *Fv*/*Fm* did not differ significantly between dark and light treatments under heat stress. The 50% re-reduction (RR50) of P700^+^ on cessation of far-red illumination was faster after light treatment than that in the dark. After exposure to 25 °C in a low light for 15 min, *Y*(*NO*) (the constitutive non-regulatory non-photochemical quenching) treated by heat stress and darkness was higher than that by heat stress and light. Overall, our results suggested that enhanced CEFs around PSI in low light could assist PSII against heat damage by maintaining the rate of PSII repair and inhibiting the non-radiative charge recombination in PSII reaction centers.

## 1. Introduction

In higher plants, photosynthesis is believed to be one of the most heat-sensitive physiological processes [1,2,3]. Photoinhibition occurs when the amount of light energy absorbed by plants exceeds their ability to photosynthesize [4]. Excessive absorbed light generates ^1^O_2_ that can damage D1 in the PSII reaction center [5]. Heat stress has also been reported to play an important role in affecting PSII. Studies have shown that elevated temperatures cause D1 protein cleavage [6,7] through accumulating reactive oxygen species (ROS) [8].

Grapevine is a woody, perennial plant with worldwide economic importance, among which *Vitis vinifera* L. is the most cultivated grapevine species. It has been found that temperatures above 40 °C could damage photosynthesis in some grapevine cultivars [9,10]. Notably, the combination of light and temperature may trigger synergistic or antagonistic effects on the photosynthetic performance, depending on the investigated species, experimental conditions, and methods applied. Under natural conditions, highlight and heat stresses are always superimposed, and PSII photoinhibition becomes apparent under high temperature (usually exceeded 40 °C) combined with a high photosynthetic photon flux density (PPFD) (exceeded 1500 μmol m^−2^ s^−1^) [11,12]. Of particular interest are some studies showing that low light can alleviate the impact of heat stress on photosynthesis, as observed in isolated spinach chloroplasts [13], leaves of pea [14], alpine plants [15], tropical rainforest trees [16], and tropical crassulacean acid metabolism plants [17]. However, the underlying mechanism(s) remains largely unclear at present [16,18,19].

Low light might alleviate the impact of heat stress on PSII by enhancing the proton gradient across the thylakoid membrane (ΔpH), hence driving additional ATP required for protein synthesis [14] and inducing non-photochemical quenching in antenna [*Y*(*NPQ*)] and/or reducing detrimental non-regulated energy dissipation [*Y*(*NO*)] in the reaction center, which consequently decreases the yield of singlet oxygen and protects PSII, according to the excess energy hypothesis [20]. High ΔpH can also slow down linear flux (LEF) via the Cyt *b6*/*f* complex and therefore protect PSI [21]. On the other hand, low light could also assist PSII against heat stress via enhancing PSII repair cycle through the suppression of ROS production [4,22]. The build-up of ΔpH depends on various electron transport pathways, mainly LEF and cyclic fluxes around PSI (CEFs). Under environmental stresses, LEF decreases due to the inactivation of downstream enzymes, leading to a critical role of CEFs in mitigating the over-reduction of the electron transport chain during photosynthetic processes. This decreases the production of ROS and subsequently protects PSII against heat stresses [23,24]. It has been observed that higher plants have two different CEF pathways: one that depends on PGR5 and PGR5-LIKE1 (PGRL1) proteins [25,26] and another that consists of a chloroplast NADH dehydrogenase-like (NDH) complex [27]. When PSI electron acceptors are limited, both the PGR5/PGRL1- and NDH-dependent pathways play a role of recycling electrons through the plastoquinone pool without accumulating reducing power even at low light intensities [25,28] and inducing energy-dependent quenching (qE) [29]. 

Currently, it is unclear whether low light can enhance repair capacity during high temperature stress. Further, it is also unknown if low light can stimulate high CEF at high temperature and assist the build-up of ΔpH, which has multiple roles in PSII and PSI protection. In this study, photosynthetic energy partitioning theory was applied to investigate the roles of *Y*(*NPQ*) and *Y*(*NO*) in light-induced PSII protection under heat stress. We also measured the post-illumination P700^+^ re-reduction kinetics and fluorescence rise to detect the occurrence of CEFs. Leaves from grapevines were sampled under dark or low light conditions, in the presence or absence of chloramphenicol, an inhibitor of chloroplast protein synthesis [30], in order to separate the resistance and repair capacities. We propose that cyclic electron transport around PSI at low light intensity functions in alleviating the negative impacts of high temperature on PSII in grape leaves. 

## 2. Results

### 2.1. Effect of Heat Stress on Photosystem Activities

**Figure 1:**
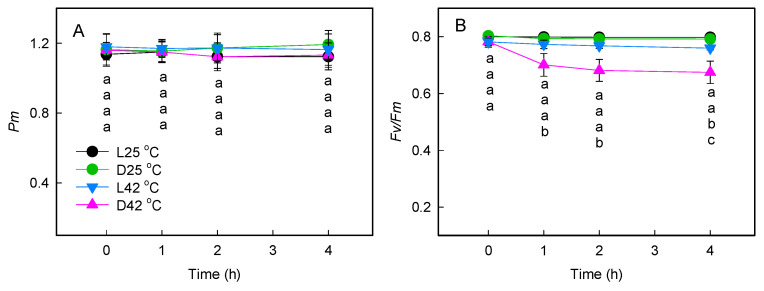
Effects of heat treatment on *Pm* (**A**) and *Fv*/*Fm* (**B**) in grape leaves. Leaves of grapevine seedlings grown at 25 °C in a greenhouse were heated for different times in the dark (D42 °C) or light (L42 °C). Leaves treated at 25 °C in the dark (D25 °C) or light (L25 °C) were used as control. Each value of the leaves was obtained after 30 min of dark adaptation. Significant differences between different temperature treatments are indicated by different letters (*p* < 0.05). Values are means (±SE) from six to eight individual plants.

**Figure 2:**
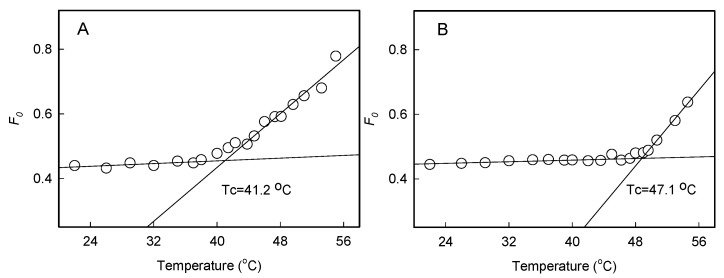
Changes in *F*_0_ upon increases in temperature with time in the dark (**A**) or light (**B**). A regression line extrapolated from the slow- and fast-rising portions of the temperature-dependent *F*_0_ response was used to calculate Tc. Values are means from three individual samples.

To explore the effects of interaction between light and heat stress on changes in the photochemical activities of PSI and PSII, we compared the maximum photo-oxidizable P700 (*Pm*) and the PSII maximum quantum efficiency (*Fv*/*Fm*) levels in the dark (D42 °C) or under 200 μmol photons m^−2^ s^−1^ light (L42 °C). No significant changes of *Pm* were observed after heat stress (42 °C) in the dark or under 200 μmol photons m^−2^ s^−1^ light (Figure 1A), which indicates that PSI activity was thermally stable at high temperatures. A striking difference was observed between leaves in darkness and those in light in response to heat stress. In the dark, *Fv*/*Fm* decreased significantly when leaves were exposed to high temperatures for over 1 h, but in the light, it changed slightly. As treatment time increased, plants in the light showed significantly higher values of *Fv*/*Fm* (Figure 1B). Furthermore, with the increase in leaf temperature, the critical temperatures (Tc) in the dark and light were approximately 41 and 47 °C based on *F*_0_ values, respectively (Figure 2), indicating that thermotolerance of PSII was enhanced in the light.

### 2.2. Effect of Heat Stress on Chlorophyll a Fluorescence Rise OJIP Kinetics

**Figure 3:**
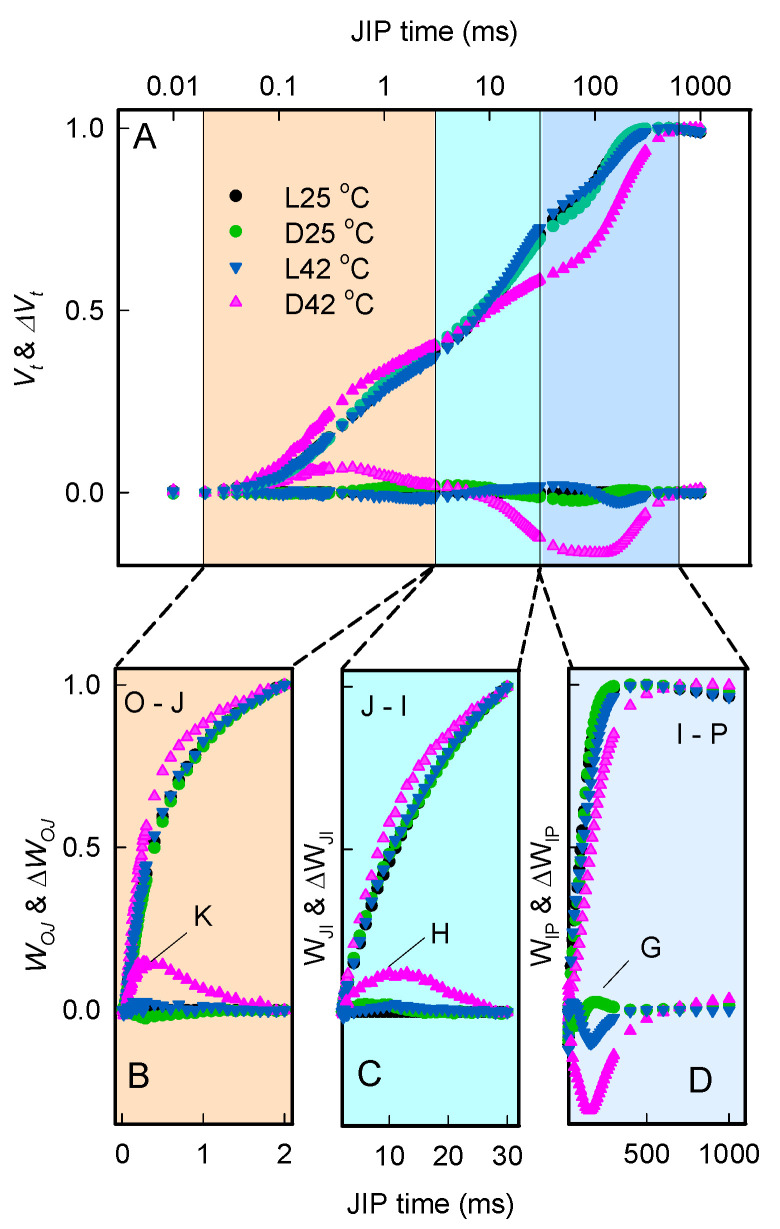
Responses of relative chlorophyll a fluorescence of leaves after heat treatment. Leaves were treated with temperature of 42 °C for 4 h before measurements. (**A**) Chlorophyll a fluorescence rise kinetics normalized by *F*_0_ and *F_M_* as *Vt* = (*Ft* − *F*_0_)/(*F_M_* − *F*_0_) (top) in a logarithmic time scale, and the difference kinetics ∆*Vt* = *Vt* (treated)−*Vt* (control) (bottom) were represented. (**B**) The fluorescence a rise kinetics normalized by *F*_0_ and *F_J_* as *W_OJ_* (top) and the difference kinetics *ΔW_OJ_* (bottom). (**C**) The fluorescence a rise kinetics normalized by *F_J_* and *F_I_* as *W_JI_* (top) and the difference kinetics *ΔW_JI_* (bottom). (**D**) The chlorophyll a fluorescence a rise kinetics normalized by *F_I_* and *F_P_* as *W_IP_* (top) and the difference kinetics *ΔW_IP_* (bottom). Each curve is the average of six independent measurements.

Chlorophyll a fluorescence rise OJIP kinetics were normalized between *F*_0_ and *F_M_* to further assess how heat stress affects the photochemical activity of PSII, and presented as relative variable fluorescence *Vt* = (*Ft* − *F*_0_)/(*F_M_* − *F*_0_) and ∆*Vt* = *Vt*(treated) − *Vt*(control) (Figure 3A, “Control” samples are those treated at 25 °C in the dark). An analysis of the differences between the *Vt* and ∆*Vt* curves of leaves showed that the treatment of heat stress in the dark had a pronounced positive K peak and negative I peak compared to that in the light.

We also normalized and subtracted the chlorophyll a fluorescence rise kinetics based on the heat stress effects on chlorophyll a fluorescence rise OJIP kinetics, reflected in the OJ, JI, IP, and OI phase (Figure 3B–D). Comparing different kinetics, *W_OJ_* and ∆*W_OJ_* between leaves treated with 42 °C proved that heat treatment provoked a positive K peak that was larger in the dark and smaller in the light (Figure 3B), indicating the inactivation of the OEC centers in treatment of D42 °C. As shown in Figure 3C, the chlorophyll a fluorescence rise kinetics were normalized between the J and I step and are presented as relative variable fluorescence *W_JI_* and ∆*W_JI_*. The curve of ∆*W_JI_* gives information about the H-band that represents the dynamics of reduction in the plastidquinone (PQ) pool. The positive values of the differences in H-bands indicated a relatively small PQ pool and suppression of its reduction as a result of heat stress in the dark. The amplitude of the G-band (30–300 ms) can characterize the PSI end electron acceptors condition. Negative values of the differences in the G-band (Figure 3D) revealed an increase in the relative size of the pool of PSI end acceptors in the leaves treated with heat stress in the dark.

### 2.3. Effect of Heat Stress on D1 Protein Level

**Figure 4:**
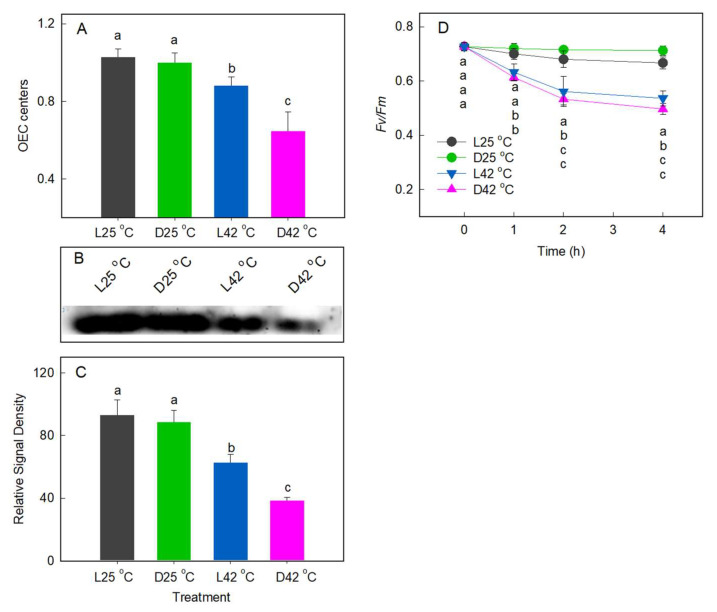
Effects of heat stress on PSII photochemical activity and D1 protein level. (**A**) The fraction of oxygen evolving complex (OEC) centers. (**B**) Changes in D1 protein levels after heat stress treatment. (**C**) Quantitative image analysis of protein levels for Figure 4B. (**D**) Changes of *Fv*/*Fm* in chloramphenicol-treated leaves. Significant differences between different temperature treatments are indicated by different letters (*p* < 0.05). Values are means (±SE) from six to eight individual plants.

After heat treatment, the fraction of the active OEC centers decreased significantly in treatment of D42 °C. However, a smaller reduction in the OEC centers was found in leaves treated with L42 °C (Figure 4A). A tyrosine residue in the D1 protein (Yz) is a redox component closely associated with OEC, and heat damage to PSII is mainly due to D1 protein degradation [31]; thus, increasing the rate of D1 protein synthesis is necessary to improve PSII repair efficiency under heat stress. To investigate whether the differences in PSII activity in response to heat stress in the dark and in the light were related to the D1 protein degradation, we performed Western blot analysis of thylakoid membrane proteins of treated leaves. The results showed that heat stress in both the dark and in the light reduced D1 protein content, but a greater decrease in D1 protein content was observed in the dark than in the light (Figure 4B,C). The results indicate that the D1 degradation affected PSII activity under heat stress. Figure 4D shows that in the presence of the D1 protein synthesis inhibitor chloramphenicol, the *Fv*/*Fm* of the leaves was progressively reduced by heat stress, but the difference of *Fv*/*Fm* between the dark and light no longer existed. Taken together, these data demonstrate that the enhanced thermotolerance of PSII in low light-adapted leaves was associated with an improvement in preventing the degradation of D1 protein.

### 2.4. Effect of Heat Stress on the Re-Reduction Rate of P700^+^ after Far-Red Illumination and the Post-Illumination Chlorophyll Fluorescence

**Figure 5:**
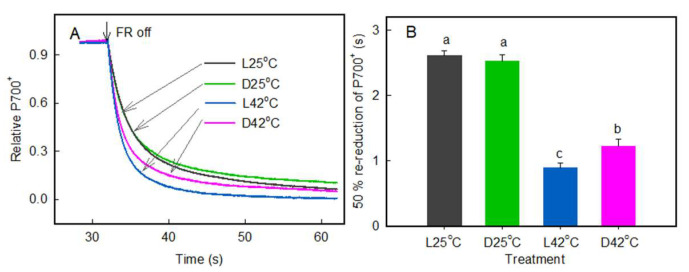
Effects of heat stress on re-reduction of P700^+^. Leaves were treated with a temperature of 42 °C for 4 h before measurements. P700 was oxidized by illumination of the leaf with far-red light (FR) for 30 s, and after the termination of far-red light illumination, P700^+^ re-reduction was monitored in the dark. (**A**) Kinetics of P700^+^ re-reduction in the dark after far-red illumination. Each curve is the average of six to eight independent measurements. (**B**) The 50% re-reduction of the P700^+^ was determined after far-red illumination. Significant differences between different temperature treatments are indicated by different letters (*p* < 0.05). Values are means (±SE) from six to eight individual plants.

The heat-induced change of the re-reduction of P700^+^ rate after a period of far-red illumination can give a measure of the PSII-independent reduction rate of PSI, which is considered to comprise CEF around PSI and electron donation from stromal reductants to the photosynthetic electron transfer chain [32]. Figure 5 shows that after far-red illumination, the oxidized reaction center of PSI (P700^+^) was re-reduced. High temperature (at 42 °C) caused a marked acceleration of the re-reduction rate of P700^+^, with 50% re-reduction (RR50) of the re-reduction curve decreased from approximate 2.5 s (at 25 °C) to about 0.9 and 1.2 s in the light and dark, respectively.

**Figure 6:**
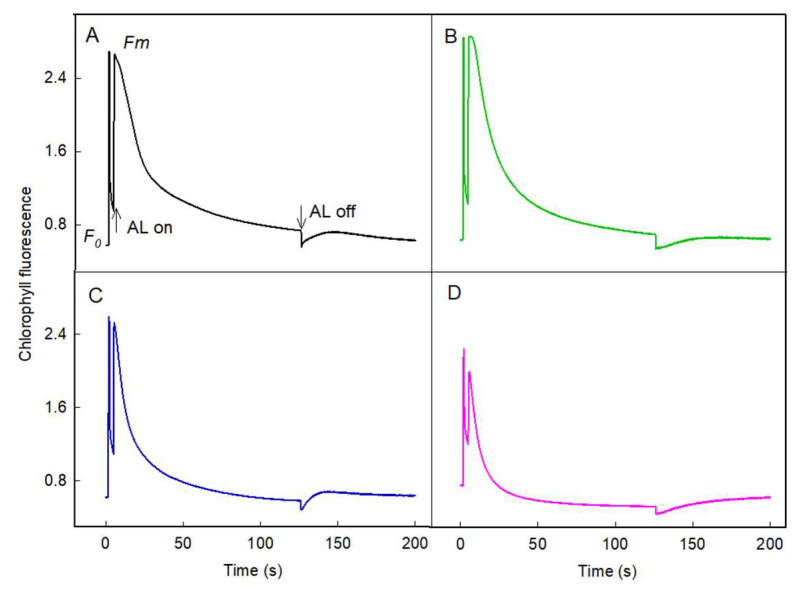
Effects of heat stress on the post-illumination chlorophyll fluorescence. Following a 2 min exposure to actinic light, changes in chlorophyll fluorescence was monitored after the actinic light was turned off. Leaves were dark adapted for 30 min prior to measuring the fluorescence. A typical post-illumination transient increase induction curve in leaves under L25 °C (**A**), D25 °C (**B**), L42 °C (**C**), and D42 °C (**D**). Each curve is the average of six to eight independent measurements.

After illumination of actinic light under normal temperature (25 °C), a transient increase in chlorophyll fluorescence both in the dark and light was detected (Figure 6A,B). When the leaves were treated with high temperature (42 °C), the magnitude of the increase became greater in the light (Figure 6C), with only a slight increase in the dark (Figure 6D), indicating that the electron flow from stromal reductants to PQ under heat stress is enhanced by light [33].

### 2.5. Energy Distribution in the Steady-State PSII following Heat Treatment

**Figure 7:**
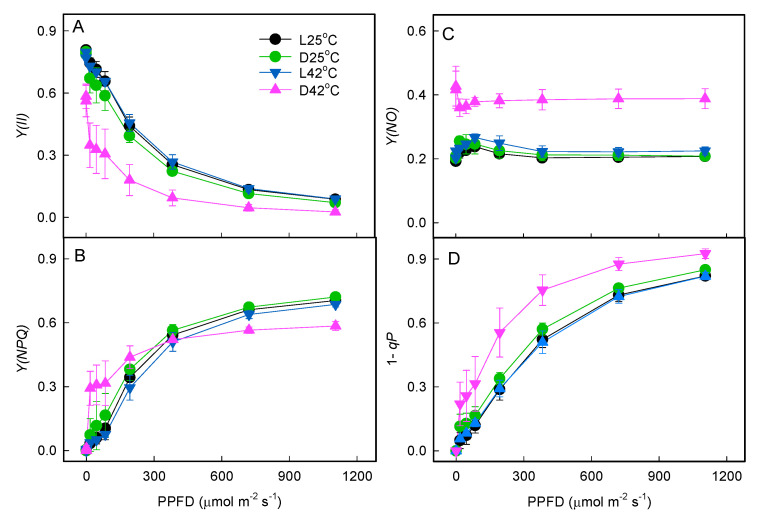
Light response of quantum yields of the steady-state PSII following heat treatment. After treatment with heat stress in the light or dark, leaves were exposed to 383 μmol photons m^−2^ s^−1^ for 15 min at 25 °C, and then *Y*(*II*) (**A**), *Y*(*NPQ*) (**B**), *Y*(*NO*) (**C**), and 1-*qP* (**D**) in light-adapted leaves were recorded. *Y*(*II*), effective quantum yield of PSII photochemistry; *Y*(*NO*), quantum yield of non-regulated energy dissipation in PSII; *Y*(*NPQ*), quantum yield of regulated energy dissipation in PSII; 1-*qP*, the fraction of closed PSII centers. Values are means (±SE) from six to eight individual plants.

After 15 min of light exposure, the PSII photochemical yields [*Y*(*II*)] in treatment of D42 °C decreased more, especially under low light on the light response curves, compared with treatment of L42 °C (Figure 7A). Meanwhile, the quantum yield of regulated energy dissipation in PSII [*Y*(*NPQ*)] in treatment of D42 °C showed a lower increase (Figure 7B). Nevertheless, the quantum yield of non-regulated energy dissipation in PSII [*Y*(*NO*)] and the fraction of closed PSII centers (1-*qP*) was much higher than other treatments (Figure 7C,D).

## 3. Discussion

### 3.1. Low Light Enhances PSII Thermostability via Repair Not Resistance Process

In the present study, our results show that PSII in grapevine seedlings became more resistant to heat stress damage when exposed to low light. Evidence included the lesser decline of *Fv*/*Fm* and higher level of *Tc* at low light compared to those in the dark. Our results also show that D1 protein content maintained a relative high level compared to that in the dark, and a more serious destruction of oxygen evolution complex (OEC) occurred after exposure to high temperature in the dark, as reflected in an obvious increase in the level of ∆*K* peak (∆*W_K_*) of the OJIP curve. The results are consistent with some studies by using other plant species and confirmed that low light can enhance PSII thermostability.

Further, by applying chloramphenicol, an inhibitor of D1 protein de novo synthesis, we found that the alleviation effect by low light was largely diminished in the presence of chloramphenicol, suggesting that low light enhances PSII thermostability via repair but not resistance process. This demonstrates the critical role of PSII repair in PSII thermostability. He and Chow [34] found the value of repair rate coefficient was near maximum at an irradiance as low as 29 µmol photons m^−2^ s^−1^ at room temperature and decreased with the increase in irradiance. This makes us further postulate that the antagonistic or synergistic effects of temperature and light on PSII performance might mainly depend on the PSII repair capacity. This result supports the viewpoint of Takahashi and Murata [35] that exposure to environmental stresses does not affect photodamage but inhibits the PSII repair through suppressing PSII protein synthesis.

Heat-stressed leaves in darkness showed lower *Y*(*II*), higher 1-*qP*, and a pronounced H-band compared to leaves at room temperature, suggesting over-reduction of the photosynthetic electron transport chain, possibly caused by the inactivation of carbon assimilation enzymes induced by heat stress. However, since PSI was not damaged in darkness, and it was found that PSI is more vulnerable when its acceptor side is over-reduced [36], it is more likely that the over-reduction of electron acceptors is located at the donor side of PSI (i.e., PQ pool). Indeed, 1-*qP* and H-band (*ΔW_JI_*) are regarded as signs of a degree of reduction in the PQ pool. Over-reduction of the PQ pool can also increase the chance of charge recombination within the PSII reaction center, as evidenced by a higher level of *Y*(*NO*). Enhanced charge recombination incurs the generation of singlet oxygen and consequently damages the PSII reaction center directly. In darkness, photochemical activities do not lead to the over-reduction of the PQ pool. The data obtained in our study showed that PQ reduction and electron donation to P700^+^ were greatly enhanced in heated leaves after exposure to dark, compared to the control. This finding evidences that alternative pathways that supply reducing equivalents to PSI from stromal reductants are substantially activated in the dark [37]. In fact, Marutani et al. [38] found that in darkness, heat stress enhances introduction of reducing power from stroma into thylakoid membranes and causes over-reduction of plastoquinone. The origin of reducing equivalents might lie in the metabolism of starch in the dark [39].

### 3.2. Low Light Enhances Cyclic Electron Flows around PSI under Heat Stress

Repair of damaged PSII needs considerable energy [40], and since *Y*(*II*) is not different between 25 and 42 °C in low light, supplementary electron fluxes coupled with ATP/NADPH are necessary to explain how low light enhanced PSII repair in the present study. Indeed, enhanced CEFs around PSI were observed when leaves were exposed to low light under heat stress, compared to the other three treatments, as evidenced by the significantly faster P700^+^ re-reduction (RR50 decreased), indicating CEF was induced by heat [41]. In this study, heat stress in the light also induced a higher post-illumination fluorescence rise level. In contrast, a decreased level of fluorescence with slower kinetics was found when leaves were heated in the dark, indicating that the electron flow from stromal reductants to PQ is enhanced under heat stress in the light [33].Previous studies have reported that the electron flow from NADPH to PQ is regulated by light in intact chloroplasts [42], and the activation of CEF (NDH-dependent pathway) in isolated thylakoids of barley is light-dependent [43]. Moreover, NAD(P)H dehydrogenase activity can be restored by light as a result of light-induced expression of subunit B but lost during the preceding dark incubation in *Synechocystis* cells [44].

It is also supposed that CEFs around PSI help to build up extra ΔpH to facilitate nonphotochemical exciton quenching (*NPQ*), consistent with the result of a higher *Y*(*NPQ*) by low light treatment than that by dark treatment when leaves were exposed to heat stress, particularly under moderate to strong actinic light conditions [45]. Further, enhancement of CEFs around PSI also mitigates the over-reduced PQ pool and hence reduces the probability of singlet oxygen generation. In this study, it is worth noting that we cannot quantify the contributions of PGR5-PGRL and NDH pathways only by post-illumination P700^+^ re-reduction and fluorescence or other possible electron transport pathways such as plastoquinonol terminal oxidase (PTOX) or the water–water cycle (WWC). Mutants (e.g., PGR5) or chemicals (e.g., 2-thenoyltrifluoroacetone) are needed in future studies.

## 4. Materials and Methods

### 4.1. Plant Material and Treatments

One-year-old self-rooted grapevine seedlings of ‘Cabernet Sauvignon’ (*Vitis vinifera* L.) were grown in plastic pots (25 cm in diameter) filled with garden earth, matrix soil, and sand (2:1:1, *v*/*v*/*v*) under greenhouse conditions. The culture conditions were 26/22 °C day/night temperatures with a maximum PPFD of about 1200 μmol m^−2^ s^−1^. Grapevine seedlings with 10 outspread leaves were chosen for heat stress treatment according to our previous methods [10]. As controls, seedlings were kept at 25 °C in the light of 200 μmol m^−2^ s^−1^ (L25 °C) or in the dark (D25 °C) for 4 h. The other seedlings were treated with high temperature (42 °C) in the light of 200 μmol m^−2^ s^−1^ (L42 °C) or in the dark (D42 °C) for 4 h.

The critical temperature (*Tc*) was determined using detached leaves [46], and was used to estimate the heat resistance of PSII in plants [46,47].

### 4.2. Measurement of Chlorophyll a Fluorescence

Chlorophyll a fluorescence OJIP transients were measured using a plant efficiency analyzer (Handy-PEA, Hansatech Instruments, Norfolk, UK). Before the measurements, leaves were dark-adapted for 20–30 min. Recorded original data include the fluorescence intensity at 50 μs (*F*_0_), the maximum fluorescence intensity (*Fm*), and the fluorescence intensities at 300 μs (K-step), 2 ms (J-step), and 30 ms (I-step). The induction curves occurring at different times were normalized for visualization of the OJIP transitions [48,49,50]: *Vt* = (*Ft* − *F*_0_)/(*Fm*−*F*_0_), *W_OJ_* = (*Ft* − *F*_0_)/(*F_J_* − *F*_0_), *W_JI_* = (*Ft* − *F_J_*)/(*F_I_* − *F_J_*), *W_IP_* = (*Ft* − *F_I_*)/(*F_P_* − *F_I_*) and *W_OI_* = (*Ft* − *F*_0_)/(*F_I_* − *F*_0_), where *W* is the relative variable fluorescence. Difference kinetics between the leaves under D25 °C conditions and other samples at OP, OJ, JI, and IP time regions were calculated as ∆*W* = *W* − *W*(*control*). The K-band (*ΔW_OJ_*), H-band (*ΔW_JI_*), and G-band (*ΔW_IP_*) are presented Figure 3.

According to the JIP test [51], the chlorophyll a fluorescence parameters were calculated: the PSII maximum quantum yield (*Fv*/*Fm*), *Fv*/*Fm* = (*Fm* − *F*_0_)/*Fm*, where *F*_0_ is the minimal chlorophyll fluorescence intensity and *Fm* is the maximal chlorophyll fluorescence intensity. The active fraction of oxygen evolving complex (OEC) centers was calculated on basis of K-step fluorescence [52]: OEC centers = [1 − (*V_K_*/*V_J_*)] treatment/[1 − (*V_K_*/*V_J_*)] control.

### 4.3. P700 and Chlorophyll Fluorescence Analysis

P700 and chlorophyll fluorescence analysis were measured by pulse amplitude modulated fluorometry (Dual PAM-100, Heinz Walz, Effeltrich, Germany). The re-reduction kinetics of P700^+^ were measured by monitoring absorbance at 830 nm according to previously described methods [53,54,55]. The 50% re-reduction of P700^+^ was calculated to indicate the occurrence of CEF. The maximal change of P700 signal (*Pm*) was determined through application of a saturation pulse after illumination of far-red light for 10 s to indicate the relative functional PSI content [56,57]. The transient post-illumination increase in chlorophyll fluorescence was monitored as described by Shikanai (1998) [55]. A typical post-illumination transient increase induction curve was recorded after termination of the 2 min illumination with active light (383 μmol photons m^−2^ s^−1^) as an indicator of CEF activity [33,58].

The chlorophyll fluorescence parameters of PSII under steady-state conditions after 15 min of AL illumination following heat treatment were calculated. The rapid light curves of chlorophyll fluorescence were determined as described by Klughammer and Schreiber [20]. The following chlorophyll fluorescence parameters were calculated [30,31]: actual PSII efficiency; *Y*(*II*) = (*Fm*′ − *Fs*)/*Fm*′, where *Fm*′ is light-adapted maximum fluorescence upon illumination with a pulse of saturating light, and *Fs* is light-adapted maximum fluorescence; the quantum yield of regulated energy dissipation of PSII, *Y* (*NPQ*) = *Fs*/*Fm*′ − *Fs*/*Fm*; the quantum yield of non-regulated energy dissipation of PSII, *Y* (*NO*) = *Fs*/*Fm*; and the fraction of closed PSII centers, 1 − *qP* = (*Fs* − *F*_0_′)/(*Fm*′ − *F*_0_′), where *F*_0_*′* is the light-adapted initial fluorescence. The parameters deduced from OJIP transient curves and parameters measured with pulse amplitude modulated (PAM) fluorometry are shown in Table S1.

### 4.4. Detection of D1 Protein

To evaluate overall D1 level, immunoblot assays of total protein extracts were performed using anti-D1 antibodies. Grape samples were homogenized with cooled isolation buffer (50 mM pH 7.8 Hepes, 100 mM sucrose, 2 mM EDTA, 20 mM NaCl and 2 mM MgCl_2_) and then centrifuged at 3000× *g* for 10 min. The sediments were in the same isolation buffer, re-centrifuged at 3000× *g* for 10 min, and then re-homogenized in protein sample buffer. A 12% polyacrylamide gradient gel was then used to denature and separate the protein samples. The proteins were then electro-blotted to PVDF membranes, incubated with antibodies raised against D1, and then visualized using the enhanced chemiluminescence method. Finally, the quantitative image analysis of protein levels was performed using Gel-Pro Analyzer 4.0 software.

### 4.5. Infiltration with Chloramphenicol

The effect of chloramphenicol on PSII photodamage was examined [59,60]. In order to inhibit D1 protein synthesis, chloramphenicol was used. The fully expanded leaves were excised from the plants and soaked in chloramphenicol solution (1 mM) quickly with a second excision in the solution, incubated for 4 h under weak irradiance at room temperature, and treated at 42 °C in the dark or light for 4 h. 

### 4.6. Statistical Analyses

The measurements were conducted on randomly selected samples. Values are the mean ± SD from 6 to 8 individual plants. The data were analyzed using SPSS 13 Software (IBM Corporation, New York, NY, USA) with one-way analysis of variance (ANOVA) at a probability level of *p* < 0.05 according to Duncan’s multiple range comparison.

## 5. Conclusions

In conclusion, the results of our study show that heat stress in the dark resulted in serious PSII damage. The enhanced CEFs around PSI in low light could assist PSII against heat damage by maintaining the rate of PSII repair and inhibiting the non-radiative charge recombination in PSII reaction centers.

## Data Availability

Data is contained within the article or Supplementary Materials.

## Supplementary Materials

The following supporting information can be downloaded at: https://www.mdpi.com/article/10.3390/plants11243537/s1, Table S1: Definition of parameters deduced from OJIP transient curves and parameters measured with pulse amplitude modulated (PAM) fluorometry.
