# Supplementary material for: Low Light Facilitates Cyclic Electron Flows around PSI to Assist PSII against High Temperature Stress
Source: Plants (Basel). 2022 Dec 15;11(24):3537. doi: 10.3390/plants11243537 (PMC9788621; doi:10.3390/plants11243537)
Supplement: Supplementary file 1 [file plants-11-03537-s001.zip › plants-2065076-supplementary.pdf]

Supplementary Materials

Table S1. Definition of parameters deduced from OJIP transient curves and parameters measured with pulse amplitude modulated (PAM) fluorometry.

**Chlorophyll a fluorescence parameters**	
$Vk = (Fk - F_0)/(Fm - F_0)$	Relative variable fluorescence at the K-step (300 μs)
$Vj = (Fj - F_0)/(Fm - F_0)$	Relative variable fluorescence at the J-step (2 ms)
$Fv/Fm = 1 - F_0/Fm$	Maximum quantum yield for primary photochemistry
$W_{OJ} = (Ft - F_0)/(F_J - F_0)$	Relative variable fluorescence at the K-step to the amplitude $F_J - F_0$
$W_{JI} = (Ft - F_J)/(F_I - F_J)$	Ratio of variable fluorescence $Ft - F_J$ to the amplitude $F_I - F_J$
$W_{IP} = (Ft - F_I)/(F_P - F_I)$	Ratio of variable fluorescence $Ft - F_I$ to the amplitude $F_P - F_I$
OEC centers = [1 − (Vk/Vj)]treatment/[1 − (Vk/Vj)]control	Fraction of oxygen evolving complexes (OEC) centers
**Chlorophyll fluorescence parameters**	
$Y(II) = (Fm' - Fs)/Fm'$	Effective quantum yield of PSII
$Y(NPQ) = Fs/Fm' - Fs/Fm$	Quantum yield of regulated energy dissipation of PSII
$Y(NO) = Fs/Fm$	Quantum yield of non-regulated energy dissipation of PSII
$1 - qP = (Fs - F_0')/(Fm' - F_0')$	Fraction of closed PS II centers
